# When the Lowest Energy Does Not Induce Native Structures: Parallel Minimization of Multi-Energy Values by Hybridizing Searching Intelligences

**DOI:** 10.1371/journal.pone.0044967

**Published:** 2012-09-28

**Authors:** Qiang Lü, Xiao-Yan Xia, Rong Chen, Da-Jun Miao, Sha-Sha Chen, Li-Jun Quan, Hai-Ou Li

**Affiliations:** 1 School of Computer Science and Technology, Soochow University, Suzhou, China; 2 Jiangsu Provincial Key Lab for Information Processing Technologies, Suzhou, China; Bioinformatics Institute, Singapore

## Abstract

**Background:**

Protein structure prediction (PSP), which is usually modeled as a computational optimization problem, remains one of the biggest challenges in computational biology. PSP encounters two difficult obstacles: the inaccurate energy function problem and the searching problem. Even if the lowest energy has been luckily found by the searching procedure, the correct protein structures are not guaranteed to obtain.

**Results:**

A general parallel metaheuristic approach is presented to tackle the above two problems. Multi-energy functions are employed to simultaneously guide the parallel searching threads. Searching trajectories are in fact controlled by the parameters of heuristic algorithms. The parallel approach allows the parameters to be perturbed during the searching threads are running in parallel, while each thread is searching the lowest energy value determined by an individual energy function. By hybridizing the intelligences of parallel ant colonies and Monte Carlo Metropolis search, this paper demonstrates an implementation of our parallel approach for PSP. 16 classical instances were tested to show that the parallel approach is competitive for solving PSP problem.

**Conclusions:**

This parallel approach combines various sources of both searching intelligences and energy functions, and thus predicts protein conformations with good quality jointly determined by all the parallel searching threads and energy functions. It provides a framework to combine different searching intelligence embedded in heuristic algorithms. It also constructs a container to hybridize different not-so-accurate objective functions which are usually derived from the domain expertise.

## Introduction

**Figure 1:**
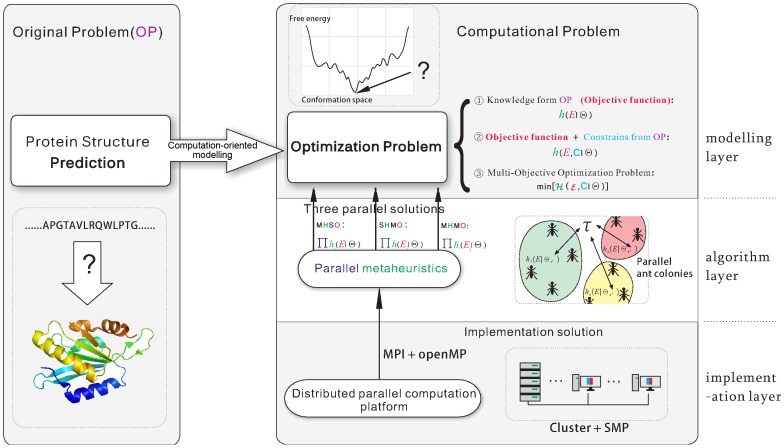
General framework of solving PSP by parallel metaheuristic. The OP stands for all kinds of application problems, which can be computationally modeled as an optimization problem. Three such models are possible for solving OP, namely 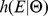, 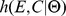 and 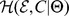, where 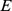 and 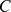 are derived from how to solve OP numerically and non-numerically respectively. Three parallel solutions can be applied to solve the modeled optimization problems, and the current parallel platform at both hardware and software level can easily support the above three solutions.

Given the protein’s amino acid sequence, protein structure prediction (PSP) is to predict the tertiary structure of its native state. It still remains one of the biggest challenges in computational biology [1], [2]. According to the hypothesis that the native structure always adapts to the status with the lowest free energy [3], PSP is usually converted to a single-objective optimization problem (SOP) which tries to minimize the free energy value of the predicted structure (see the left part of Figure 1). Such problem and its variants have been proved as NP-hard problems [4], [5]. Therefore, metaheuristic [6] is a natural choice to tackle PSP problem (see the modeling layer of Figure 1). There are two biggest major obstacles for solving PSP problem [1], [7]: the first is that the searching is always inefficient even if the current computing power is increasing exponentially, and the second is that as the objective function for minimizing, energy function itself cannot accurately measure the free energy of a computer-generated conformation because we are lack of complete knowledge on measuring free energy based on protein conformation surrounded by the complex bio-environment. The first obstacle introduces an intrinsically hard problem in computing fields, while the second must address how to accurately calculate the free energy in biology fields.

Energy functions, such as physical energy functions, empirical potentials, and even those based on the approximations of quantum chemistry, are usually derived from the analysis of the properties of protein in different views and levels. Unfortunately, all existing energy functions are inaccurate in a universal sense, but each of them is very useful in some specific senses. This hypothesis is referred to as the *inaccuracy/usefulness* property in this paper. An energy function typically consists of a combination of weighted energy terms. The parameters and constants for evaluating the energy of a specific term are usually derived from empirical studies or theoretical hypothesis. From the viewpoint of the *inaccuracy/usefulness* property, all the energy terms in the various energy functions are correct in a qualitative sense for determining which kinds of interactions should be taken into account when evaluating free energy. If an energy function can discriminate native structure or its legal equilibrium from computer-generated conformations, this energy function is with usefulness, otherwise with inaccuracy. It is easy to confirm that when being applied to the native structures available on the Protein Data Bank (PDB) [8], any calculative energy function is of usefulness for some native structures and of inaccuracy for other native structures. Similar real experimental justifications of *inaccuracy/usfulness* property can be found in such as [9], [10].

We tend to accept that the native structure always adopts the lowest free energy [3], and that such correct free energy function cannot be accurately calculated right now. What we have to use are various approximations of free energy functions. So it is natural to raise this question: when the lowest energy value of a must-use energy function does not induce native structures, what should we do?

The simplest approach is to integrate different sources of energy functions. A typical solution is to use a support vector machine (SVM) to regress the outputs of several energy functions [11], [12]. In this way, the inaccuracy of some energy functions can be complemented by the usefulness of other energy functions. Alternatively, trying to train new parameters with coarse-grained pattern is another approach to tackling the *inaccuracy/usefulness* of the original energy function [13]. However, the new regressed or fitted energy function still has the *inaccuracy/usefulness* property because the new function is basically just another linear combination of the original energy functions. Such approaches adopt SOP as the computational model for solving PSP (see case 1 in the modeling layer of Figure 1).

Since a single energy function is not enough for solving PSP, multi-objective optimization problem (MOP [14]) seems more reasonable as the computational model for better modeling PSP. However, because of the *inaccuracy/usfulness* property of energy functions, solving PSP is far more complicated than solving a specific MOP. An MOP itself is very hard in computational view [14], [15], not to speak of an MOP with inaccurate objective functions to be optimized for PSP.

If the accurate free energy can hardly be computed to discriminate the native or near-native structures from computer generated structures, it is more reasonable to model a PSP problem as an MOP (see case <$>\scale 70%\raster="rg1"<$> in the modeling layer of Figure 1) so that more considerations of energy function can be brought in together in order to find discriminative free energy values. Instead of the lowest values of multiple objective functions, Pareto front (PF) [15] is used in order to evaluate the quality of a solution for an MOP.

A multi-objective evolutionary approach to the PSP problem has been proposed in [9], where CHARMM energy function was segmented into two parts, bond and non-bond items. So the PSP problem is converted to a two-objective minimization problem. Using a GA-based heuristic algorithm, this method evolves the population (a collection of solutions) and constructs the PF by evaluating the solution with the two energy functions. Finally the predicted structure is selected by finding the most sensitive solution from the PF. Five proteins with 34–70 residues were tested in [9]. Another two simple MOP solutions have been applied to PSP [16], [17], where the similar technique was used to build PF and three simple energy items were simultaneously minimized for four testing proteins.

For either SOP or MOP, computing power is mostly not enough. In general, parallel metaheuristic [18] is a good choice for such optimization problems. Parallel tempering is a very classical parallel metaheuristic with a lot of applications in physics, chemistry and biology [19]. The basic idea of parallel tempering is to exchange the ongoing best-so-far solutions (named replicas in the literature) of the parallel sampling procedures in a scheduled timing. Such replicas are generated by parallel search processes ruled by different parameters, such as simulated temperatures. By accepting the replica which is optimized by the other search process with different search parameters, the search process is able to keep on optimizing the intermediate result coming from the other search process. Similarly, ant colony optimization (ACO) [20]–[22] is also a very popular metaheuristic straightforward for parallelization. Different search paradigms have been intensively studied for parallel ACO algorithms [23]. The key issues of studying parallel ACO are what kind of information should be exchanged among the cooperative agents (ants or colonies), and how to design the exchange such as to whom and when the information should be exchanged [24]–[26]. Most of the parallel ACO implementations have been applied to different SOPs, for example, traveling salesperson problem (TSP) and quadratic assignment problem [27], time and space assembly line balancing problem [28], [29], multi-depot vehicle routing problem [30], and protein-ligand docking problem [31], [32] (They named their system with PLANTS (Protein-Ligand ANT System)). A very interesting thing is that PLANTS also suffers very much from the inaccurate energy functions [33], where two different energy functions have been developed and identified for different categories of docking problems. A recent study of PLANTS is to port the calculation of energy function to graphics processing units [34]. The literature of parallelization ACO studies shows that little has been done on applying ACO to MOP for solving PSP.

It is not difficult to ensure that parallel technologies are good to solve PSP. A parallel Rosetta based on OpenMP [35] implementation has been developed by partitioning Rosetta’s prediction protocol into parallel running procedures, and the parallel predictions have been evaluated in four protein cases [36]. A massively parallel strategy has been applied to an old benchmark task which run half day in a 65536-CPU cluster [37]. All these studies have featured building a virtual huge CPU from the parallel CPU cores. The above two studies are trying to solve PSP problem with SOP model in parallel metaheuristic ways. Besides, the prediction protocol running in parallel remains unchanged compared with its sequential version, and therefore the prediction accuracy has not been improved substantially.

The approach of combining both MOP and parallel metaheuristic to PSP was first found in [10]. A loose computing grid was conducted for a GA-based solver of PSP based on MOP model, and two testing predictions on that platform demonstrate the power of the parallel computing [10]. In general, parallel metaheuristic is an interesting research topic in terms of application of heuristic algorithms, and the evaluation of parallel multi-objective evolutionary algorithms is also a very difficult problem [38].

In this study, a novel parallel approach is proposed to combine the *usefulness* and decrease the *inaccuracy* of different energy functions. The parallel approach makes ACO searches and Monte Carlo search run in parallel and exchange their searching intelligences. Multiple energy functions are employed by the parallel search threads. Thus, both the searching and energy knowledge can be hybridized to obtain the predictions. As for the *inaccuracy/usefulness* problem, the major difference between the parallel solution proposed here and other solutions is that the energies of our method are minimized under nondeterministic guidance by parallel threads which are constrained by multiple different energy functions.

## Methods

A general parallel metaheuristic framework is first described in this section, and a parallel scheme of the general framework is then introduced to solve PSP problem.

### General Parallel Metaheuristic Framework for PSP

We propose a general framework for solving PSP problem by parallel metaheuristics, illustrated in the right part of Figure 1.

For modeling target original problem (OP), the PSP or other kind of similar OPs can be converted to an optimization problem, as shown in the modeling layer of Figure 1. Three computational models which usually rely on metaheuristic technologies are used to solve such optimization problem.

1. Single objective model. We admit that the best answer to OP can be judged by an existing objective function 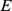. We denote a heuristic algorithm for solving SOP by 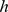. So solving OP means solving 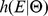, where 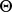 refers to the control parameters in terms of heuristic search algorithm and can usually be tuned empirically before starting the algorithm or adaptively during the algorithm is running. For example, for a Monte Carlo search with Metropolis criterion [39], the temperature can be considered as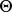. Another more complicated example of 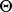 is the pheromone matrix of ACO algorithm, see details after describing three parallel schemes later. Different designs and settings of 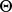 will result in different searching trajectories when sampling the search space of SOP. Hence 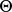 can be considered as the searching intelligence of 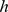 in some degree.
2. Single objective with constrains model. Some knowledge in terms of solving OP is not easy to be represented as a numerical metric in the forms of energy 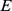. Instead, the knowledge can guide to restrict the search trajectory in a form of programming logic. We denote such constrains by 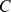. So with this model, solving OP means solving 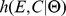. The reason why we consider 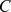 independent from 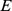 is that we do not enforce that all the solving knowledge of OP must be encoded as an energy function.
3. Multi-objective model. If different energy functions 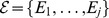 and heuristics 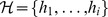 have to be used to solve OP, it is very easy to derive such computational model: 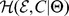. This is a typical MOP model with multiple heuristics involved.

We shall remind that even the simplest model, SOP model 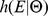, is still a hard problem for computing scientists.

In the algorithm layer of Figure 1, we propose three parallel schemes to tackle all the above modeled optimization problems.

1. MHSO, Multi-Heuristic-Single-Objective algorithms. This type of algorithms can be designed to solve both 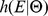 and 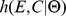 models. If we assume that 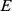 is accurate enough to identify the best answer to OP, we can then employ different search heuristics 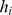
s to run in parallel. During the running, 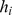
s exchange their searching experiences by sharing (some of) the control parameters 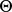. This type of algorithms is denoted by 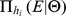, and focuses on combining different heuristics to solve an SOP problem as the common 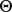 carries intelligences coming from parallel heuristic algorithms.
2. SHMO, Single-Heuristic-Multi-Objective algorithms. If different kind of energy functions 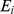
s must be taken into consideration while an excellent search heuristic 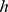 is preferable, we design to run several same 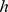
s in parallel each of which can work with different 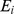
s. Again all the parallel 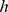
s are partly controlled by the common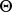, which will be simultaneously updated by 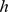 during its running for locating the lowest 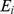 value. This type of algorithms is denoted by 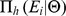, and focuses on combining different objective functions to solve an MOP problem because different objective functions affect parallel heuristic search trajectories by dynamically updating 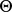.
3. MHMO, Multi-Heuristic-Multi-Objective algorithms. It is easy to understand that this scheme is the combination of the above two, denoted by 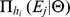. MHSO focuses on combining different search intelligences of heuristic algorithms. SHMO enables different solving knowledge from OP to affect differently on the same search policy. MHMO is aimed at hybridizing all the search intelligences and solving knowledge from target domains.

All the above three types of algorithms have the same property: the search intelligence 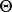 created by the parallel 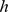
s is shared and perturbed among all the running 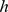
s. So the key point here is how to represent 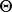 for publishing the search intelligence to parallel searches. For ACO algorithms, it is straightforward to represent 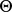 with pheromone matrix. The ACO metaheuristic is a framework generalized by a set of successful ant algorithms [40]–[42]. The ant algorithm was inspired by the observation of how ants within a colony find the shortest path in a cooperative way [43], [44]. Basically each ant tries to choose the shortest path based on both its own senses (heuristics in ACO terminology) and its ancestors’ good experience (pheromone in ACO terminology). While an ant is finishing its path, it leaves its own pheromone along the trajectory, and the subsequent ants will sense the pheromone. The best so far solutions found by ancestor ants can be segmented. The pheromone matrix describes the goodness of segments accumulated by previously found solutions. Ant algorithms are a simple and efficient method for demonstrating swarm intelligence for optimization problems [45]. For our parallel design, it is very helpful to adopt pheromone matrix as the representation of 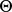.

In the implementation layer of Figure 1, with the help of either MPI [46] or OpenMP [35] all the three parallel schemes are not difficult to implement.

Strictly speaking, all the three parallel schemes are not enough for solving a standard MOP because they do not focus on building PF. But they target well for solving PSP due to *inaccuracy/usefulness* hypothesis. See Discussion section for details.

### An MHMO Implementation for PSP

An SHMO algorithm was previously implemented in order to solve PSP [47]. In that paper, 8 parallel ant colonies, which followed MMAS [42] algorithmic design and shared one pheromone matrix, were introduced to tackle CASP8/9 FM problems [47]. We named that system with pacBackbone. In this study, we design an MHMO version for solving PSP to show how to combine search intelligences of different heuristic algorithms. We add a new heuristic algorithm to the parallel ant colonies with hybridized searching intelligences between heuristics. In order to raise the difficulty of optimization problem, we focus on de novo PSP problems, which means that few experimentally solved structures could be found for predicting protein as homologs. Without losing generality of illustrating our method, we only consider the backbone prediction problem which is a prerequisite task of PSP. In order to clearly demonstrate the parallel power of MHMO scheme, we skip those processes too specific for CASP8/9 problems, such as loop rebuilding and weighting scores for clustering decoys which were essential parts for goals of ref. [47]. We call the system presented in this paper pacBackbone+.

#### Problem representation

The backbone is represented as a sequence of torsion tuples 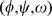, where each tuple represents spatial information on a residue. All atoms of each side-chain are simplified as a single pseudo-atom. Two fragment libraries from the Robetta online server [48], 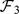 and 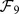, are used for each target amino-acid string to sample torsion tuples. For each of the residues within the query target chain, each fragment library provides 200 3-mer and 9-mer fragments. Each segment is assigned one of the predicted secondary-structure (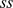) labels H, E, or L.

Because the 3-mer and 9-mer fragments are coarse-grained fragments which focus mainly on capturing H and E secondary-structure features, 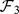 and 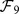 are merged to form 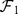, which contains a 1-mer torsion tuple for each residue. 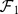 is used to refine these non-H/E segments after the backbone has been roughly constructed. In this way, 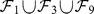 defines the search space for predicting the target backbone.

Based on the above descriptions, the problem of de novo prediction of a protein backbone is to find the structure of protein backbone with the lowest energies for an amino-acid sequence within a fragment-based search space after filtering out those fragments covered by the sequence’s homologies. This problem can be formulated as follows:

#### Problem

Given an amino-acid sequence 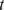 with length of 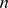 residues, predict its backbone with the lowest free energy.

#### Search space

Let fragment library be 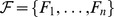 for 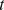, where fragment set 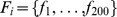 for each residue 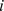 in 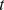. Fragment 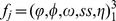 is the 3-mer fragment for a residue, where 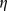 denotes similarity measure between segments. Such fragments form a 3-mer fragment library 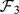. Similarly, fragment set 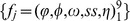 forms a 9-mer fragment library 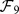. 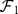 is the local search space which is constructed by collecting all 1-mer fragment of 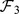 and 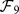. So the search space 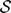 for target 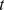 is 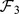 and 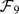 for each 3/9-residue segment, and 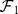 for each residue.

#### Energy function

We adopt the same energy functions as what Rosetta de novo prediction protocol developed [49]. Rosetta3.2 protocol uses 5 energy functions (score0, score1, score2, score5 and score3) at different stages of the predicting procedure [50]. Each stage contains a lot of Monte Carlo movements filtered by Metropolis criterion. At the final stage of prediction, Rosetta protocol minimizes the energy of the conformation with score3. In fact, these scores are the combinations of different weights and energy items, such as residue-environment and residue-residue interaction, secondary structure packing, chain density and excluded volume [49], [51]. Table 1 lists the detailed weights for combining the different energy items used by Rosetta.

**Table 1:** Different scores used as the minimizing objective functions.

energy items	score0	score1	score2	score5	score3	memo
env	0.00	1.00	1.00	1.00	1.00	residue environment
pair	0.00	1.00	1.00	1.00	1.00	residue pair
cbeta	0.00	0.00	0.25	0.25	1.00	C 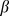 density
vdw	0.10	1.00	1.00	1.00	1.00	steric repulsion
rg	0.00	0.00	0.00	0.00	3.00	radius of gyration
cenpack	0.00	0.00	0.50	0.50	1.00	residue packing
hs_pair	0.00	1.00	1.00	1.00	1.00	helix-strand packing
ss_pair	0.00	0.30	1.00	1.00	1.00	strand pair
rsigma	0.00	0.00	0.00	0.00	1.00	second-structure interaction
sheet	0.00	1.00	1.00	1.00	1.00	strand arrangement
ss_lowstrand	0	1	1	1	0	not effective
ss_cutoff	0	11	6	11	6	not effective

The design of applying different scores at different prediction stages in Rosetta inspires us that MHMO might be a valuable way of solving PSP.

We shall emphasize that we do not care which score function is more accurate here because all the energy functions share the *inaccuracy/usefulness* property in this study. Adopting energy functions of Rosetta here is just for illustrating an implementation of our parallel approach, and for comparing the results in Section Results. Of course another reason for choosing Rosetta as the control platform for results comparison is that Rosetta has been successfully validated as a super platform for PSP and the consequent applications [52], [53].

#### Applying multiple heuristics and energy functions to PSP

**Figure 2:**
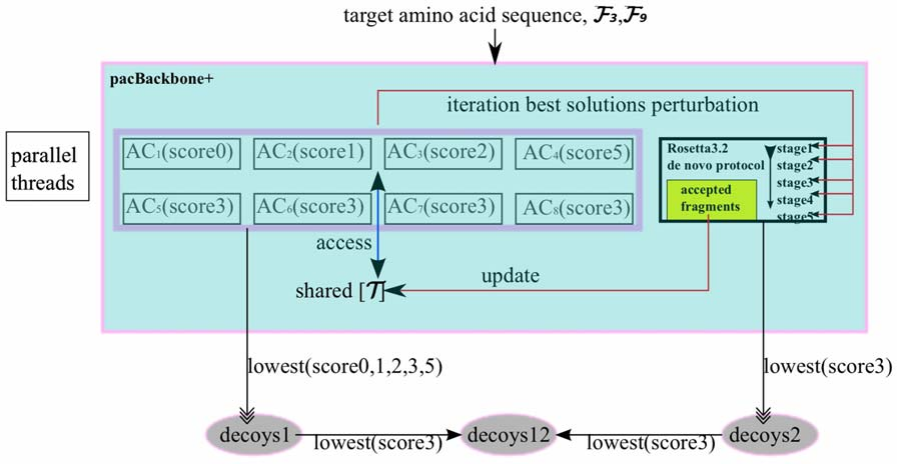
pacBackbone+ schematic flowchart. Two different heuristics are introduced, 8 ant colonies and 1 Rosetta predictor are running in parallel threads. The colonies share one pheromone matrix 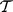, and Rosetta predictor sends accepted fragments to AC colonies for updating 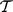. Also AC colonies send the iteration best solutions to Rosetta to perturb the conformation at every beginning of the prediction stage. The information exchanged between AC colonies and Rosetta predictor is colored by red feedback lines, and the information exchanged among AC colonies is colored by the blue line.

9 parallel threads are created in our MHMO implementation. 8 of them are ant colonies and the rest is a modified Rosetta3.2 de novo predictor [50] which was based on Monte Carlo search filtered by Metropolis criterion. Figure 2 depicts the design of pacBackbone+. The inputs of pacBackbone+ are target amino acid sequence and 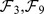 generated based on the target sequence. The outputs are decoys predicted by ant colonies and Rosetta predictor, denoted by decoys1 and decoys2 respectively.

**Figure 3:**
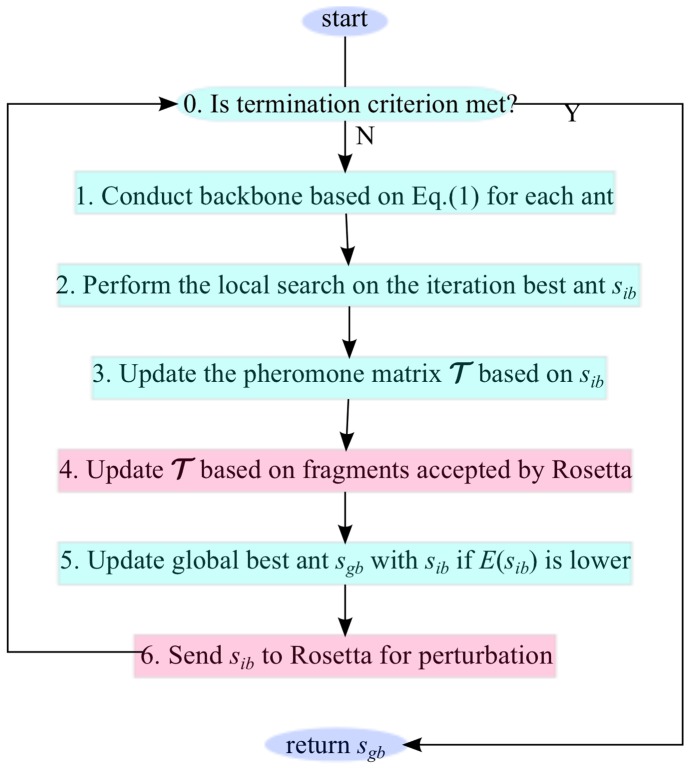
Single ant colony AC searches the lowest 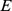 with shared 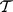. A standard MMAS algorithm with perturbation from and to Rosetta predictor each other. The blue parts depict the original MMAS components (0, 1, 2, 3, and 5), and the pink parts depict the interaction between AC and Rosetta predictor (component 4 and 6).

Figure 3 illustrates a single ant colony for predicting the backbone with constraints from a single energy function. We now explain each component of this figure.

The first important data structure of the AC is the pheromone matrix 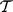, which accumulates the search experience of all ants in the colony. The next important input of the AC is the energy function 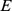. All ants cooperate iteratively to search for the overall best backbone 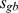 with the lowest 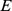 value.

In component 1 of Figure 3, each ant conducts the conformation by assembling fragments from 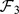 and 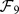. First, each ant chooses 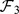 with a preset probability 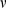, and 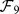 with 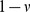. 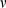 is an algorithm parameter to tune the preference of the choice between 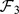 and 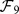. Then the ant picks up a fragment 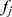 from the fragment set 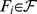 for residue 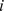. How to select fragment is determined by the current heuristic and historical knowledge, described by the following selection equation:
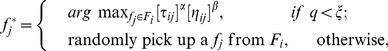
(1)where 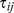 is defined later in equation (2), which denotes the useful experience accumulated by previous searches. 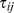 gives a bias clue for choosing segment 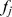 for residue 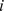. Within the ACO framework, 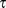 denotes a pheromone, and 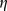 denotes heuristic information. 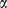 and 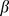 are standard ACO parameters which tune the assigned weights of the heuristic and the pheromone. Similarly to 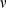, 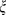 in equation (1) serves to tune the bias between the two selection policies. A random probability 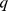 will be first uniformly generated when a fragment is needed. If 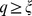, then the fragment will be randomly picked from 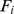. Equation (1) is called selection equation in ACO framework, and has various criteria [40]–[42]. The current form of selection Equation (1) is proved to be most successful in the ACO literature [20]. Once the fragment is picked, 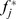 is inserted into the peptide from the position of residue 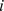. The conformation is then constructed by the approach called fragment assembly. Each ant conducts the conformation by repeating such fragment picking and assembling for 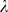 times.

In component 2 of Figure 3, the iteration best conformation 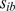 is fed to a local search algorithm which tries to improve 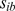 with some local movements. The paper uses a one-flip strategy [54] combined with Metropolis criterion to implement the local search. The approach is to replace one fragment with one randomly selected from 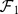 to obtain better quality in terms of the whole conformation. Such replacement is accepted by the Metropolis criterion [39].

Another important component of AC, shown in component 3 of Figure 3, is to update the pheromone matrix 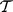 after all ants have finished their assembly work in an iteration. The pheromone matrix stores the search knowledge collected from the ants of previous iterations. Let the pheromone matrix be: 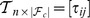, where 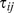 is the pheromone value accumulated by residue 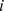 picking up fragment 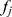. For each residue 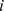 in 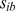,
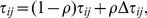
(2)where 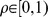 is the evaporation factor of the pheromone. Let 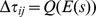, where 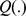 is the quality function which converts the energy value to a certain amount of pheromone. In this paper, the function used is 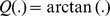, so that the pheromone value is scaled to 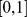. Different update schedule derives different ACO algorithm. Here we use MMAS [42], one of the best ACO algorithms, as the pheromone update mechanism.

pacBackbone+ introduces another source to update 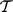, shown in component 4 of Figure 3. A modified Rosetta predictor is running in parallel with the colonies, and produces a sequence of accepted fragments by its own criterion. This sequence is kept by pacBackbone+, and used by the 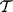 update procedure. After updating 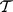 with 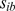, pacBackbone+ uses the new produced sequence of fragments to update 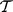. pacBackbone+ considers these fragments coming from the choice of the Rosetta “best-so-far-ant”.

The iteration of construction and updating activities terminates when certain criteria are met, shown in component 0 of Figure 3. Following the advice summarized in [55], each colony AC terminates when one of the following criteria is met:

1. Soft time criterion: the colony runs for a specified number of iterations.
2. Hard time criterion: the colony runs with a specified maximum CPU running time.
3. Convergence criterion: successive iterations indicate a convergent state, such as no energy improvement during the last ten iterations.
4. Search space criterion: the search space has been covered to a certain extent, such as more than 50%.

In this paper, we choose the first criterion as the termination criterion, which is to set a maximum iteration number to run the colony.

After describing AC parts of Figure 2, we now introduce Rosetta predictor thread in Figure 2. Rosetta3.2 de novo protocol for predicting protein backbone sequentially uses five energy functions at different predicting stages, score0 at stage1, score1 at stage 2, score2/score5 at stage 3, and score3 at stage 4 and 5 [50]. At all the earlier four stages, Rosetta assemblies fragments based on 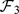 and 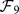, and at the final stage makes independent random perturbations of torsion angles of residue. In pacBackbone+, we modified the original Rosetta3.2 predictor to make it to communicate with AC colonies. The iteration best solutions of AC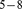 (PerturbationSet) are stored for perturbing Rosetta predictor at the beginning of every stage, (see also component 6 of Figure 3). The average value 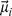 of the torsion angles for each residue 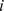 of the PerturbationSet is calculated. For the current working conformation of Rosetta, we modify the torsion angles 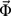 of each residue with the average value of the PerturbationSet like this:
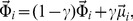
(3)where 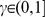 is a parameter for adjusting the perturbation degree from AC colonies. So we enforce Rosetta predictor to be affected by the results from AC searches. Vice versa, the accepted fragments by Rosetta predictor are sent to AC colonies for updating 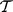.

The implementation of our MHMO scheme is simple with the help of OpenMP [35]. The pheromone matrix is extracted from the AC, and multiple colonies are run as parallel threads with private data in each colony except for the pheromone matrix 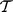.

## Results

The experimental results here serve a proof-of-concept validation of a parallel design, MHMO scheme. We performed all the tests on a computer cluster containing 20 nodes with 16-core 1.9 GHz AMD Opteron CPU per node under Linux 2.6.9 and GCC 4.3.3. pacBackbone+ and pacBackbone were all implemented upon Rosetta3.2 platforms [50].

### Experimental Setting

We ran the original sequential Rosetta3.2 de novo predictor to produce 800 predictions for each test instance as the control experiment. Rosetta’s predictor is based on a sequential Monte Carlo search with 5 energy functions involved in different predicting stages. We named the results of the sequential Rosetta predictor with decoys0. We ran pacBackbone+, which had 8 ant colonies and 1 modified Rosetta predictor running in parallel, on the same test instances. Because we assigned 8 CPU cores to AC colonies and 1 CPU core to the modified Rosetta predictor and all these 9 threads were synchronized to work out 9 predictions respectively, 800 predictions for each test instance (named decoys1) were generated by AC colonies and 100 predictions (named decoys2) by the parallel Rosetta predictor. The final results of pacBackbone+ was named decoys12, which had 800 predictions with lower score3 values of the union of decoys1 and decoys2 (see Figure 2). In order to identify whether hybridizing two different heuristics helps improve the accuracy of the prediction, we also ran pacBackbone [47], which had only 8 parallel AC colonies without interaction with Rosetta predictor, on the same test instances, and named the results with decoys3. In a word, all the decoy sets have 800 predictions for each test instance except decoys2 having 100 predictions. decoys0 is the results of sequential running, and others are those of parallel running. In this study, we evaluated the quality of the decoys based on statistical analysis of both the decoys population and the representative prediction, since how to select most near native structure from the decoys is not a concern of this paper.

The benchmark instances were directly from [7], which contained 16 small protein targets with length of 49–88 amino acid residues. The performance evaluation between two prediction methods appears to be another difficult problem. Several factors would affect the performance of prediction algorithm, such as computational representation of protein, dihedral angle space, energy function, folding strategy and test sets [56]. We carefully constructed the comparative experiment to demonstrate how pacBackbone+ was working well on this classical benchmark. The settings of the control factors for compared systems were configured equivalently, including the search space, the multiple energy functions, the running time and the comparable algorithm parameters settings.

The fair evaluation of different heuristics is always a difficult task. When the calculation of energy function is a time-consumed job, the number of calling energy functions can be usually used to compare the efficiency of the search trajectories. However, in this paper such approach is not suitable for our case due to different strategies which the compared heuristics adopt. AC colony adopts population-based evolvement strategy, which carefully constructs a whole conformation by each ant of the colony and then evaluates the conformation. Meanwhile, Rosetta’s predictor uses individual-based evolvement strategy, which makes local change to the current individual conformation and evaluates the conformation right once. This means that Rosetta’s predictor usually makes quick decision to evolve the only one conformation and hence frequently calls energy functions, while AC colonies spend much efforts to build multiple conformations within colony before they call energy functions. Therefore, we adopted another reasonable strategy to set the base line in terms of running time for our performance evaluation.

Now we explain how to fairly assign CPU cost to each of the decoy sets, which turns out bias towards the control experiment. The sequential Rosetta3.2 predictor, compiled as an executable file named minirosetta.gcclinuxrelease, was run with the same input options for each problem instance:

in:file:native/path/to/native pdb file
in:file:fasta/path/to/target fasta file
in:file:frag3/path/to/3-mer fragment library
in:file:frag9/path/to/9-mer fragment library
out:pdb true -abinitio::increase_cycles 4 -out::nstruct 800
run:protocol abrelax
mute all
database/path/to/rosetta32-database

As this was the setting for the control set decoys0, all other settings for compared systems were referred to this base line. First, for decoys3, we adjusted the algorithm parameters to make each prediction spend strictly less CPU time than that of decoys0. Then for decoys1 we used the same settings as decoys3. In this way, we enforced two different heuristics (AC and Rosetta) to spend almost same CPU time for doing a prediction. Due to the synchronization restriction between AC colonies and modified Rosetta predictor, even same settings for decoys1 and decoys3 resulted in different CPU time. Decoys1 spent a little bit more time than decoys3 because of the extra synchronization cost with decoys2. Finally, the almost same CPU time of decoys1 was assigned to decoys2. This was done by tuning increase_cycles a little bit bigger than the setting for decoys0. Decoys12 almost spent nothing additionally as it was just a simple combination of decoys1 and decoys2. So in terms of CPU time, decoys12 = max (decoys2,decoys1)
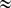
decoys3
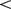
decoys0.

Next we describe how to set AC-specific parameters for generating decoys3 and 1. As for the AC colonies, given that 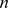 is the residue number, the algorithm parameters were set empirically: probability 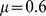 for selecting 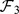, 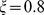 and 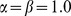 in equation (1), 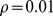 in equation (2), 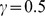 in equation (3), the termination criterion in Figure 3 was set to 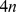 iterations, and the tries number 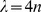 for each ant of assembling fragments. Finally the ant number of a single colony was set to 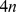.

### Running Time Comparison

We list all the exact running time in Table 2. The average running time of decoys0 is expressed in seconds for obtaining one prediction of the decoys. The time of decoys1, 2 and 3 is expressed as a ratio number, which is the running time of the corresponding decoys to that of decoys0. The time of decoys12 is the maximum of decoys1 and decoys2. From Table 2 we can see, we assigned strictly less CPU time to decoys3 than to the sequential version (see also the previous section). This means that the parallel AC colonies (pacBackbone) did not get more CPU time than the sequential Rosetta’s predictor. Since decoys3 and decoys1 shared the same algorithm parameters, theoretically they should spend the same CPU time. But in fact, decoys1 had to spend additional time to accept the perturbation from Rosetta’s predictor (updating pheromone matric based on the fragments picked by Rosetta’s predictor). As a consequence, such synchronization time of parallel running made decoys1 spend more 24% time than decoys3. Even with such non-algorithmic cost, decoys1 and 3 spent almost less time than decoys0. It is interesting to notice that decoys2 spent less time than decoys0 although decoys2 took bigger increase_cycles setting. Because the increase_cycles controls the number of Monte Carlo trials of Rosetta’s predictor, bigger increase_cycles usually spends larger CPU time. Analyzing the log message from decoys2 revealed that it was the AC colonies’ perturbation who shortened the stage 3 of the Rosetta’s prediction procedure. The stage 3 was converged shortly after it had received best predictions from AC colonies. That was why decoys2 took less CPU time than decoys0 although the former took more Monte Carlo trials.

**Table 2:** The comparison of average running time for obtaining each prediction.

PDB ID	Residue§	decoys0(s)†	decoys1‡	decoys2‡	decoys3‡	decoys12‡
1af7_	72	158.52	1.25	0.98	0.98	1.25
1b72A	49	101.04	0.51	0.48	0.44	0.51
1csp_	67	227.31	1.28	1.17	0.93	1.28
1dcjA_	73	274.59	1.22	1.17	0.87	1.22
1di2A_	69	135.95	0.98	0.77	0.97	0.98
1dtjA_	74	171.41	1.03	0.75	0.96	1.03
1mkyA3	81	213.53	1.16	0.84	0.75	1.16
1mla_2	70	192.77	0.82	0.64	0.71	0.82
1nouA4	69	162.77	0.96	0.79	0.83	0.96
1o2fB	77	181.44	1.10	0.87	0.80	1.10
1ogwA_	72	185.07	0.84	0.66	0.63	0.84
1r69_	61	101.82	1.06	0.85	0.68	1.06
1shfA	59	156.39	0.56	0.48	0.42	0.56
1tif_	59	151.90	0.61	0.57	0.48	0.61
1tig_	88	226.70	1.55	1.01	0.94	1.55
2reb_2	60	102.32	0.95	0.79	0.61	0.95

§ Residue number of each test instance.

† The average running time of decoys0 is expressed in seconds for obtaining one prediction of the decoys.

‡ The time of decoys1, 2 and 3 is expressed as a ratio number, which is the running time of the corresponding decoys to that of decoys0. The time of decoys12 is the maximum of decoys1 and decoys2.

As we focused on the performance improvement of AC colonies, the running time of decoys1 and 3 was basically fair to that of sequential running, given that decoys3 spent strictly less time than decoys0 while decoys1 shared exactly same algorithmic parameters with decoys3.

### Prediction Accuracy Comparison

For each decoy set, we eliminated half predictions with higher score3 values, which results in 400 predictions remained in decoys0, 1, 12, and 3, and 50 in decoys2. In this way, we simplified the task of selecting most near native prediction from decoys.

**Figure 4:**
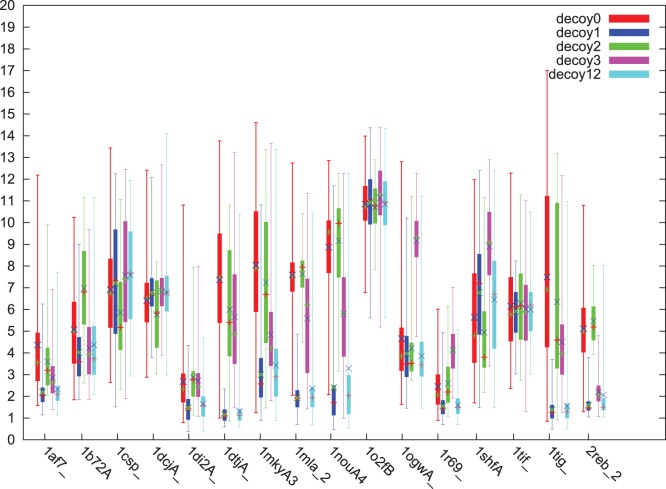
Box-and-whisker plot of all the decoys. The maximum, the minimum, the 1st quartile, the 3rd quartile, the mean (in symbol +), and the average (in symbol ×) of Ca_rmsd (Y axis in Å) of each decoy set are rendered as box-and-whisker plot for each test instance (X axis).

We first compared the prediction accuracy in terms of decoy population. We depicted the comparison of decoys with box-and-whisker plot in Figure 4. The prediction accuracy is expressed as the Ca root-mean square deviation (Ca_rmsd) in 0.1 nm (Å), which is calculated after superimposing the corresponding alpha-Carbon coordinates of the prediction and the native structure. From Figure 4, it is easy to draw a rough conclusion that the decoys1 had better performance than any other competitors. The only exceptions was for 1dcjA in decoys1. It might be caused by the special property that the case was with several beta strands connected by loops, and the search trajectory of AC was less suitable for sampling beta strands than Rosetta. However, for this only exceptional case, decoys2 showed more robust results than decoys0. Hence, it seems safe to conclude that for each test instance, there existed at least one result of parallel execution better than that of the sequential running. For the cases such as 1af7_, 1di2A, 1dtjA, 1mkyA3, 1mla2, 1nouA4, 1r69, 1tig and 2reb2, decoys1 had dominant advantage over decoys0.

**Figure 5:**
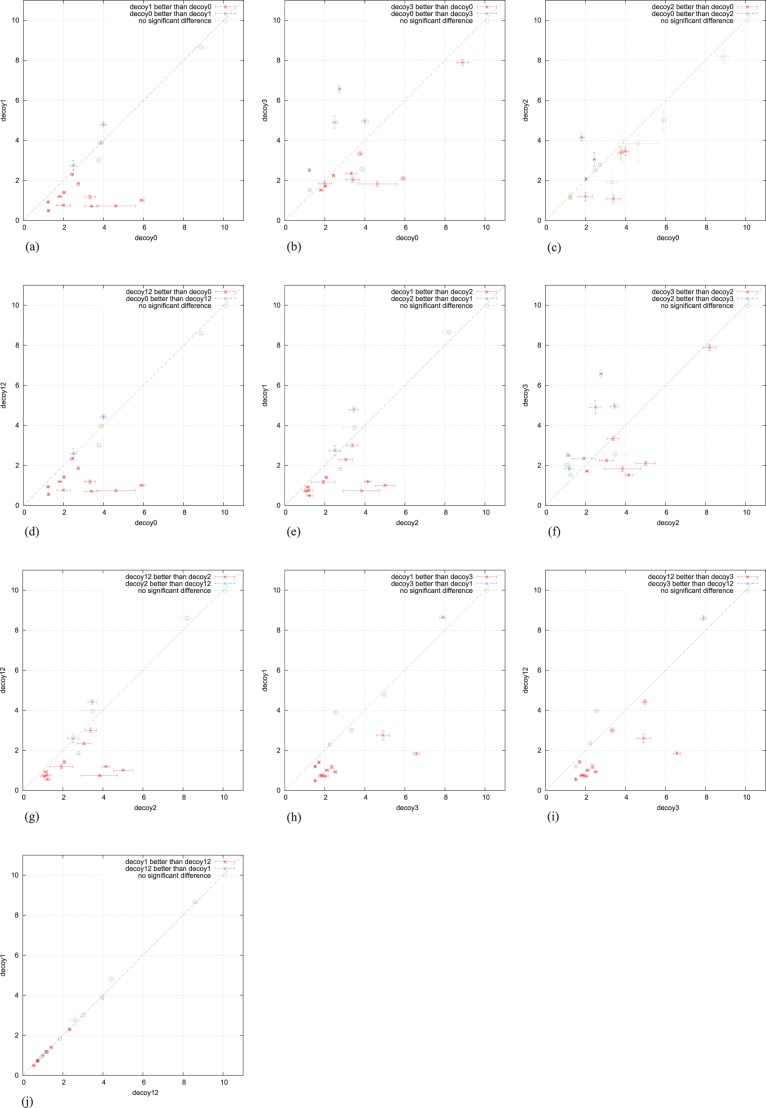
The average and standard deviation comparison of 10-percentiles of each pair of decoys. Each symbol stands for a single test instance. The standard deviation is marked as error bar, and the average is at the cross point of two error bars determined by compared decoys represented in X and Y axis respectively. For instances colored by green no significant difference has been observed.

We also performed the significance test on every pair of decoys, and results are shown in Figure 5. From Figure 5 (c) we know, that most cases of decoys2 (9 of 16) had no significant differences with decoys0, which means that the parallel Rosetta predictor had not much positive gains from AC colonies. Only 2 exceptional cases from decoys1 had no significant difference with decoys0, see Figure 5 (a). Combined with the following analysis of the comparison, it is consistent to say that parallel implementations, especially for decoys1, had better performance than the sequential one.

Next we investigated the statistical property of the representative solution of each decoy set. For each pair of decoys, we show the 10-percentiles average and standard deviation for every test instance in Figure 5, calculated by 50-fold bootstrap estimation with bioshell package [57]. The reason we choose 10-percentile as the representative is that only part of the decoys will be usually sent to further processing. The current decoy set contains backbones of protein, each of which is a low resolution structure. For a complete PSP protocol, the low resolution structure will be further refined with the high resolution (restoring all atoms of each centroid) which might cause backbone adjustment. Usually several hundreds low resolution predictions will be selected to the further processing. In our case, the 10-percentile means the 40th best prediction. So it is reasonable to evaluate the property of 10-percentiles of decoys as the indicator performance of the decoys.

From Figure 5 (a)(b) we can see, the parallel results of AC colonies (decoys1,3) were overall better than the sequential results (decoys0). For the two variants of AC parallel versions, decoys1 behaved a little bit better than decoys3 although for much of the test instances no significant difference had been observed (Figure 5 (h)). But the parallel Rosetta predictor did not show too much advantage over its sequential version (Figure 5 (c)), which implies that the parallel Rosetta predictor did not gain much positive information from AC colonies. For the comparison between the two PSP solvers, pacBackbone and Rosetta predictor, parallel AC colonies performed better than Monte Carlo method (Figure 5 (a)(b)(e)(f)). As the simple union of decoys1 and 2, decoys12 showed the average performance of two different PSP solvers (Figure 5 (d)(g)(i)). It is not surprising that decoys12 and 1 showed almost same performance because decoys2 did not contribute too much to decoys12. It seems that more complicated combination of decoys1 and 2 is needed. For example, structural diversity might be introduced instead of just using lower score3 values of the union of decoys1 and 2.

In summary, with almost fair computing time the parallel AC colonies obtained better performance than Rosetta de novo predictor, one of the stat-of-art PSP solvers, in prediction accuracy.

## Discussion

The SHOP (SHaring One Pheromone matrix) strategy was proposed as a useful parallel ACO method [58], in which multiple ant colonies execute in parallel with a single shared pheromone matrix. These multiple colonies can exchange their search experiences asynchronously and co-evolve towards better solutions while each colony is guided by its own objective function and algorithm parameters. Using ACO as the heuristic algorithms, SHOP has been easily applied to all three parallel schemes in Figure 1. For TSP, two ACO algorithms, MMAS and ACS [59], were applied to work together on the same objective function [58]. This is an MHSO implementation. Some SHMO implementations were proposed for learning Bayesian networks [60], for folding 2D proteins based on an HP model [61], and for predicting protein backbone [47]. These SHMO solutions adopted MMAS and multiple objective functions for solving different OPs. All these studies employed the same type of heuristic algorithm, ACO.

In this paper, we include another type of heuristic algorithm based on the SHOP method. An MHMO scheme of our parallel metaheuristic framework for PSP is implemented in this study, which makes parallel ant colonies and Rosetta de novo predictor coordinate with each other. These ant colonies simultaneously minimize different energy functions respectively while Rosetta predictor sequentially minimizes the same energy functions by Monte Carlo search with Metropolis criterion. This research has shown that intentional design and implementation of a parallel computing system for PSP can greatly address the problem of how to integrate the domain knowledge for solving PSP problem, and the problem of how to combine the searching intelligences for solving an optimization problem. Different knowledge for solving PSP is encoded as different energy functions, and different searching intelligences are expressed as different heuristic algorithms.

In terms of solving MOP, pacBackbone+ follows the same idea of AMALGAM [62] in putting two components into effect: simultaneous multi-method search and self-adaptive offspring creation. AMALGAM manages several GA-based algorithms to merge the strengths of parallel search algorithms by hybridizing the population generated by different searches. pacBackbone+ also makes 8 ACO-based searches and 1 Monte Carlo search run in parallel. The parallel colonies adjust the behavior of constructing backbone through the shared pheromone matrix which is perturbed by the colonies and as well as by the external Monte Carlo search. The Monte Carlo search also accepts the influences coming from the temporally best solutions of ant colonies. In terms of applying multiple heuristics to solving PSP problem, pacBackbone+ implements the similar idea of ensemble learning [63], in which replica found by different sample strategies is exchanged in a fixed schedule. Despite cooperating with an additional heuristic algorithm, pacBackbone+ is similar with multi colony ant algorithm, such as [27]. However, pacBackbone+ is critically different from the literature studies in information exchange between parallel searches. Usually the information exchanged between parallel searches is the solution or replica itself, while pacBackbone+ sends solutions from colonies to Rosetta predictor, and receives fragments (components of the solution) from Rosetta predictor. Moreover, between colonies, pacBackbone+ does not exchange solutions directly, but does exchange search intelligences encoded in 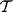. This feature differentiates pacBackbone+ from the existing parallel ACO implementations [23], [24], [26].

Under the hypothesis of *inaccuracy/usefulness* property, neither SOP nor MOP is an ideal computational model for PSP. This means even if you solve the modeled SOP or MOP completely, the final answer to the SOP or MOP might not be the right answer to PSP, not to mention that the final answer itself will not likely be the best answer to the SOP or MOP in most cases. pacBackbone+ proposes a novel hybrid parallel approach to PSP.

From perspective of solving PSP problem based on MOP, pacBackbone+ differs in not constructing PF based on multiple energy values. Pareto-based approach to MOP focuses on the dominance analysis of the solutions found by individual search or parallel searches in order to construct PF. However, this is not right in the case of solving PSP problem because PF is not the right answer to PSP. pacBackbone+ gives up constructing PF because we think that the MOP is just an approximate computational model for solving PSP problem. Instead, pacBackbone+ collects all the ¡°best¡± solutions found by different parallel searches guided by different energy functions. The key point here is that the best solution predicted by each colony in fact has been influenced by not only its own energy function but also energy functions of other colonies in a qualitative way through adjusting the shared pheromone matrix. Those best solutions are not discriminative for selecting most near native conformation by one specific energy function. The decoys will then be clustered by structural similarity so that the final predicted structure is determined by the representativeness in the decoys in terms of structural criterion. This process is implicitly consistent with the fact that the evaluation of free energy must count in entropy of conformations. How to select most near native structure from decoys is another difficult problem, which is not discussed in the paper because it is not a job for the optimization. However, improving the quality of decoys as a whole of course will be very helpful for solving the selection problem.

Now let us explain why the pacBackbone+ approach is a totally different way of combining energy functions from those sequentially hybridization approaches. From the view of an individual colony 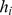, the pheromone matrix accumulates the search experience of ants. The pheromone matrix describes which fragment should be preferably considered as the choice for each residue on behalf of 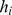. Such an empirical bias is gradually established by evaluating the conformations found by the previous generation of ants using the corresponding energy function 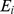. By sharing 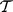 among all the colonies, especially considering that each 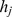 releases its pheromone trained by its own energy function 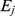, now 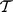 accumulates the search experience of all parallel ant colonies and propagates the bias among them. Recalling how 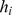 makes decisions on which fragments should be chosen based on 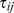 in Equation (1), the pheromone 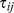 is now jointly accumulated by all the other 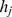
s running in parallel, not only by 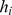 alone. Another source affecting 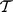 is coming from Rosetta predictor. Rosetta predictor picks fragments by the Monte Carlo search with Metropolis criterion. When the colony updates 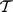, the picked fragments from Monte Carlo search are also sent to generate pheromone just as those coming from the best solution constructed by a virtual ant. Because the pheromone release procedure is not synchronized for all the parallel colonies, it is not possible to determine when to update the global 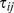 according to Equation (2). Such an indeterminacy (only in terms of a specific colony 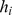) in the pheromone updating procedures is allowable or even desirable because it provides another source of randomness. Therefore, the pheromone matrix 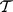 provides a nondeterministic bias for all the running colonies because of the unpredictable nature of their parallel running trajectories. We claim that pacBackbone+ is so-called a non-deterministic approach to dealing with SOP or MOP, because even given the same random seed as the randomness source, rerunning pacBackbone+ will possibly result in a different output. However, the analytical description of how the different 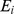
s jointly determine the ants’ performance within each colony has not been achieved yet. The most difficult issue is how to analyze the randomness introduced by the parallelization. This is also a feature which pacBackbone+ differs from the existing parallel ACO implementations. Thus the schedule of the exchange information is non-deterministic.

### Conclusions

In summary, the design of parallel metaheuristic, like pacBackbone+, not only speeds up the computation due to more CPUs being employed, but also makes each heuristic search work with its own energy function and complement each other in qualitative way. Such co-evolvement with guide of multiple objective functions mimics simultaneous impacts of the nature folding procedure of native proteins. Different energy functions train search trajectory to obtain different search intelligences, embedded in 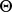. Our parallel strategy publishes the intelligence to all the parallel searches. Therefore, all searches can share the hybridized intelligence accumulated by them. Unfortunately, the quantitative analysis of such fusion cannot be reached because of the nature that the parallel threads run in unpredicted trajectories. Therefore the update of 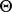 is non-deterministic. Such non-deterministic evolvement provides a novel solution to the one of the biggest obstacles of solving PSP, which is all the energy functions are not accurate but useful. Compared with the traditional experimental way to solve protein structure and the sequential or SOP way to predict the structure, the parallel approach proposed in this paper presents an interesting “*in silico* experiment” approach.
